# E-Cigarettes: Use, Effects on Smoking, Risks, and Policy Implications

**DOI:** 10.1146/annurev-publhealth-040617-013757

**Published:** 2018-01-11

**Authors:** Stanton A. Glantz, David W. Bareham

**Affiliations:** 1 Center for Tobacco Control Research and Education and Department of Medicine, University of California, San Francisco, California 94143, USA; Stanton.Glantz@ucsf.edu; 2 Lincolnshire Community Health Services NHS Trust, Louth, LN11 0EU, United Kingdom; david.bareham@live.co.uk

**Keywords:** smoking initiation, smoking cessation, cancer, cardiovascular disease, lung disease

## Abstract

Since e-cigarettes appeared in the mid-2000s, some practitioners, researchers, and policy makers have embraced them as a safer alternative to conventional cigarettes and an effective way to stop smoking. While e-cigarettes deliver lower levels of carcinogens than do conventional cigarettes, they still expose users to high levels of ultrafine particles and other toxins that may substantially increase cardiovascular and noncancer lung disease risks, which account for more than half of all smoking-caused deaths, at rates similar to conventional cigarettes. Moreover, rather than stimulating smokers to switch from conventional cigarettes to less dangerous e-cigarettes or quitting altogether, e-cigarettes are reducing smoking cessation rates and expanding the nicotine market by attracting youth.

## INTRODUCTION

Cigarettes are a highly effective way of delivering the addictive drug nicotine. They do so by burning tobacco to create an aerosol of ultrafine particles that carries nicotine deep into the lungs, where it is rapidly absorbed, then travels through the left heart, reaching the brain in a few seconds. The combustion process also generates carcinogens, oxidizing agents, and other toxins. Like cigarettes, electronic cigarettes (e-cigarettes) create an inhaled aerosol of ultrafine particles that rapidly delivers nicotine to the brain. In contrast with cigarettes, however, e-cigarettes generate the aerosol by heating a liquid, usually consisting of propylene glycol or vegetable glycerin, nicotine, and flavoring agents, without any combustion (53).

Some in the health community, particularly in England, have embraced e-cigarettes as a safer alternative to conventional cigarettes and an effective way to stop smoking conventional cigarettes (85, 105) and have approved of their use by pregnant women (118). Despite the fact that a puff on an e-cigarette is almost certainly less toxic than a puff on a conventional cigarette, this optimistic scenario has not developed. Rather than encouraging smokers to switch from conventional cigarettes to less dangerous e-cigarettes or quitting altogether, e-cigarettes are reducing smoking cessation rates and expanding the nicotine market by attracting low-risk youth who would be unlikely to initiate nicotine use with conventional cigarettes.

## TYPES OF E-CIGARETTES

E-cigarettes as originally marketed in 2004, known as cig-a-likes, were developed in China as a less dangerous alternative to conventional cigarettes (53). The early devices looked like a conventional cigarette, often including a small light on the tip that lit when the user puffed (Table 1). These early systems were generally inefficient at delivering nicotine, in part because the particle sizes of the aerosol were too large to penetrate deep into the lungs. Newer versions feature replaceable or refillable reservoirs and rechargeable batteries that generate smaller particles and more efficient nicotine delivery. These refillable systems allow users to separately purchase the e-cigarette liquid (known as e-liquid or e-juice) that contains varying levels of nicotine and comes in many different flavors (150). Running at a higher power (temperature) not only increases nicotine delivery, but also increases the amount of formaldehyde and other aldehydes that are naturally produced by heating up propylene glycol or vegetable glycerin (73, 98) and other toxins produced in the e-cigarette aerosol.

While some practitioners, researchers, and policy makers viewed e-cigarettes as a disruptive technology (122) that would compete with the established multinational cigarette company brands, by 2014 all the major multinational tobacco companies had entered the e-cigarette market. They did so either by buying existing e-cigarette companies (including Ruyan, the original Chinese e-cigarette company, which was bought by Imperial Tobacco) or by developing their own products (128). Indeed, as part of a larger policy to keep people using recreational nicotine rather than stopping tobacco use (8, 74), Philip Morris had developed the technology of the modern e-cigarette by the mid-1990s (38). As with their other alternative nicotine delivery systems, they chose not to take the product to market to avoid attracting the attention of the US Food and Drug Administration (FDA) and possibly triggering regulation of conventional cigarettes (8, 38). Although there continue to be independently owned “vape shops,” from economic and political perspectives the e-cigarette business is now part of the traditional tobacco industry (33, 78).

## WHY PEOPLE USE E-CIGARETTES

In the United States and many other countries, e-cigarettes are not subject to the same marketing and promotion restrictions that apply to cigarettes (136). As a result, e-cigarette companies are permitted to advertise on television and in mass media as well as through newer channels such as the Internet. US e-cigarette marketing expenditures increased from $3.6 million in 2010 to $125 million in 2014 (136), which translated into rapid increases in youth e-cigarette use (discussed below). Marketing messages echo well-established cigarette themes, including freedom, good taste, romance, sexuality, and sociability as well as messages claiming that e-cigarettes are healthy, are useful for smoking cessation, and can be used in smokefree environments. These messages are mirrored in the reasons that adults and youth cite for using e-cigarettes.

### Adults

Adults cite predominantly three reasons for trying and using e-cigarettes: as an aid to smoking cessation, as a safer alternative to conventional cigarettes, and as a way to conveniently get around smokefree laws (99, 116, 131). Importantly, most adults who use e-cigarettes continue to smoke conventional cigarettes (referred to as dual users). In 2014 in the United States, 93% of e-cigarette users continued to smoke cigarettes (99), 83% in France (6), and 60% in the United Kingdom (131).

### Youth

Although initial discussions within the health community about e-cigarettes focused on the potential for adults to use them as an alternative to cigarettes, youth have rapidly adopted them. In addition to the same three motivations that adults have cited for using e-cigarettes (52, 110), youth are attracted by e-cigarettes’ novelty (52), the perception that they are harmless or less harmful than cigarettes (20, 52, 109, 110), and the thousands of flavors (5, 72, 136) (e.g., fruit, chocolate, peanut butter, bubble gum, gummy bear, among others).

**Figure 1:**
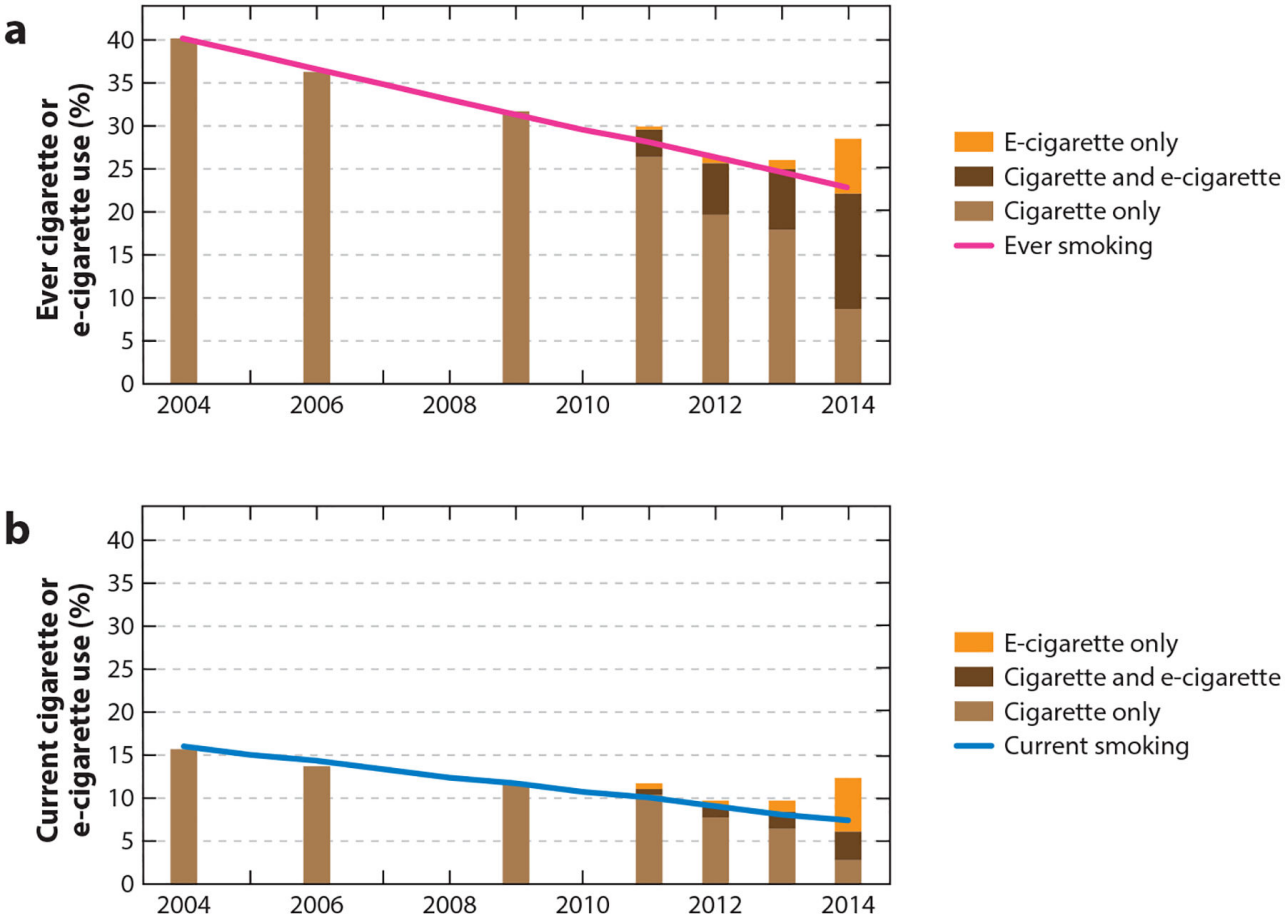
The advent of e-cigarettes did not affect declining trends in conventional cigarette smoking. After e-cigarettes became available, dual use of cigarettes and e-cigarettes increased, and some youth started using e-cigarettes alone; however, these changes did not affect the declining trend in cigarette use. This pattern was observed in both ever (≥1 puff lifetime; *panel a*) and current (use in past 30 days; *panel b*) cigarette use in the National Youth Tobacco Survey (NYTS), including dual use with e-cigarettes (cigarettes only, *light brown*; dual use, *dark brown*). E-cigarette-only users (*orange*) are at low risk of having initiated tobacco products with cigarettes (37). E-cigarette use was assessed starting in 2011. Adapted with permission from *Pediatrics* 2017 Volume 139, Issue 2, pii: e20162450. doi: 10.1542/peds.2016–2450, Copyright © 2017 by the American Academy of Pediatrics.

As a result, youth e-cigarette use in the United States doubled or tripled every year between 2011 and 2014, and by 2014, e-cigarette use had surpassed conventional cigarette use in youth (36, 117). At the same time that e-cigarette use was increasing, cigarette smoking among youth declined (9, 68), leading some to suggest that e-cigarettes were replacing conventional cigarettes among youth (1, 80, 130) and are contributing to declines in youth smoking (84, 108, 111, 123). At least through 2014, however, e-cigarettes had no detectable effect on the decline in cigarette smoking among US adolescents (37) (Figure 1).

Whereas most of the youth who reported smoking cigarettes in the past 30 days (including dual users of cigarettes and e-cigarettes) in 2011–2014 have demographic and behavioral risk profiles (based on 2004–2009 data) consistent with smoking cigarettes, the risk profiles of the remaining e-cigarette-only users (about 25% of e-cigarette users) suggested that these individuals would have been unlikely to have initiated tobacco product use with cigarettes (37). These national results are consistent with regional US studies that also found that e-cigarette-only users display a lower risk profile than do cigarette smokers for smoking cigarettes (14, 24, 93, 143). Consistent with this statement is that, in 2015, in the United States, 40% of 18–24-year-old current e-cigarette users had never smoked conventional cigarettes (27).

This rapid increase in e-cigarette-only use among youth and young adults is of concern because youth are more susceptible to developing nicotine dependence than are adults (136). In addition, nicotine has adverse effects on brain development, including that of developing fetuses (41, 134, 136).

## E-CIGARETTES AS A GATEWAY TO CIGARETTE SMOKING

A national cross-sectional study of Korean adolescents based on 2011 data was the first evidence that e-cigarette use was associated with higher cigarette use in youth (77). As with adults, dual use was the dominant pattern. The odds of being an e-cigarette user were 1.58 times [95% confidence interval (CI) 1.39–1.79] higher among students who had made an attempt to quit than for those who had not. It was rare for students who had formerly smoked but were no longer using cigarettes to be current e-cigarette users [odds ratio (OR) = 0.10; 95% CI 0.09–0.12]. A subsequent US cross-sectional study of data collected in 2011 and 2012 found similar results (35). As in Korea (77), current cigarette smokers who had ever used e-cigarettes were more likely to intend to quit smoking within the next year (OR = 1.53; 95% CI 1.03–2.28) but were less likely to have stopped smoking (OR = 0.24; 95% CI 0.21–0.28). The same US study also found that e-cigarette use was associated with progression from experimentation with cigarettes to established smoking. Among cigarette experimenters (youth who had smoked at least 1 puff of a cigarette), ever e-cigarette use was associated with higher odds of becoming an established smoker (smoking 100 cigarettes; OR = 6.31; 95% CI 5.39–7.39) and with current cigarette smoking (smoking 100 cigarettes plus smoking in the last 30 days; OR = 5.96; 95% CI 5.67–6.27).

**Figure 2:**
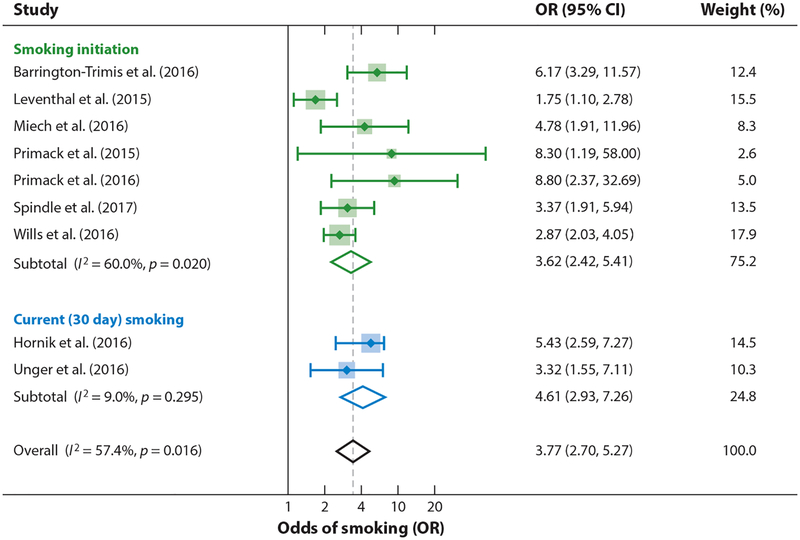
Ever e-cigarette use among never smokers at baseline quadruples the odds of being a smoker at follow-up. Meta-analysis is by the authors following Soneji et al. (119). Citations for studies: 15, 65, 79, 88, 102, 103, 121, 133, 142. Note: Weights are from random effects meta-analysis. Abbreviations: CI, confidence interval; OR, odds ratio.

Such cross-sectional data, however, do not allow investigators to draw causal conclusions because they represent a snapshot in time that does not reveal whether the e-cigarette or the conventional cigarette use came first. Reaching a causal conclusion requires longitudinal data where the same people are followed over time. As of February 2017, 9 longitudinal studies were quantifying the effect of starting tobacco use with e-cigarettes on progression to smoking conventional cigarettes (119). These studies all started with youth who had never smoked a cigarette, then compared subsequent smoking between youth who did and did not use e-cigarettes at baseline. Adjusting for demographic, psychosocial, and behavioral risk factors for cigarette smoking, the odds of subsequent cigarette smoking were quadrupled among e-cigarette users (Figure 2).

In sum, e-cigarettes are expanding the tobacco epidemic by bringing lower-risk youth into the market, many of whom then transition to smoking cigarettes. The 2015 US National Youth Tobacco Survey (117) suggests that this process may be starting. The small decline in smoking among middle-school students between 2014 and 2015 (2.5% to 2.3%) and the small increase in smoking among high school students (9.2% to 9.3%) are consistent with the observation that youth who initiated nicotine use with e-cigarettes (i.e., in 2014) are more likely to be smoking cigarettes a year later.

## E-CIGARETTES AND SMOKING CESSATION

Determining how to assess the effects of e-cigarettes on smoking cessation has been one of the most contentious aspects of the debate over e-cigarette use. In contrast with nicotine replacement therapy, e-cigarettes are mass-marketed recreational consumer products; they are not medicine developed to be administered under clinical supervision. Another issue embedded in the debate over the assessment of e-cigarettes and smoking cessation is whether their effects should be assessed only among people who are actively using them as part of a smoking cessation attempt or on all smokers who use them regardless of motivation. This situation is further complicated because a major reason that smokers use e-cigarettes is to continue inhaling nicotine in locations where conventional cigarette smoking is prohibited (e.g., workplaces, public places such as restaurants and bars, and smokefree homes) (99, 116, 131). Smokefree environments both motivate and support quit attempts (43, 95, 144, 148). By potentially dulling the effects of smokefree environments, the real-world use of e-cigarettes could reduce quit attempts and keep people smoking. As more jurisdictions include e-cigarettes in their smokefree policies and people include them in voluntary smokefree home rules, this effect will likely be diminished.

As of June 2017, there was only one prospective randomized controlled trial of people using e-cigarettes to quit smoking (23). This trial, conducted in New Zealand, compared giving patients nicotine and non-nicotine e-cigarettes with giving them a voucher for nicotine replacement therapy (NRT) that they could redeem at a local pharmacy (usual care in New Zealand). There was no significant difference in efficacy compared with nicotine patches; both patches and e-cigarettes showed low efficacy. At 6 months, verified abstinence was 7.3% with nicotine e-cigarettes, 5.8% among those offered NRT, and 4.1% for those with non-nicotine e-cigarettes. However, because participants were handed the e-cigarettes and only given a voucher for NRT, these results likely overstated the efficacy of e-cigarettes and understated the efficacy of well-managed NRT. Another randomized trial (25) that compared nicotine and non-nicotine e-cigarettes found no consistent difference in smoking cessation. This study did not have a control group of smokers not using e-cigarettes, so it does not provide any information about the effects of e-cigarette use per se on smoking cessation.

These two studies (23, 25) have been the subject of four meta-analyses (40, 58, 71, 87), two from the Cochrane Collaborative (58, 87), which concluded, with low confidence, that nicotine e-cigarettes were associated with marginally more quitting than non-nicotine e-cigarettes. Another meta-analysis (107) pooled the data from these two trials, two cohorts, and two cross-sectional studies and reached the same conclusion. None of these meta-analyses drew conclusions about the efficacy of e-cigarettes versus other interventions for cessation because only one of the trials had a non-e-cigarette comparison (control) group (23).

Most research on the relationship between the use of e-cigarettes and quitting has been from observational studies that compare cigarette use among smokers who use e-cigarettes with smokers who do not use e-cigarettes. Although it does not support the same kind of causal conclusions that an experimental study (i.e., a randomized controlled trial) would, this approach has the advantage of quantifying the effects of e-cigarettes as actually used, including any indirect effects, such as discouraging cessation attempts. An analysis of 8 cohort observational studies suggested a possible reduction in quit rates with the use of e-cigarettes compared with no use of e-cigarettes (OR = 0.74; 95% CI 0.55–1.00) (40).

Kalkhoran & Glantz (70) took a different approach, namely including all 20 available studies that reported a quantitative estimate of the association between e-cigarette use and having stopped smoking (2 clinical trials, 15 cohort studies, and 3 cross-sectional studies as of April 2015) and that had an appropriate control group (70). [They also presented a systematic review of all 38 available studies, regardless of whether they included the information necessary to estimate the effect of e-cigarette use on smoking cessation (70).] Odds of quitting cigarettes were 28% lower in those who used e-cigarettes compared with those who did not use e-cigarettes (OR = 0.72; 95% CI 0.57–0.91). This conclusion did not significantly depend on differences in the study designs: studies of all smokers using e-cigarettes (irrespective of interest in quitting cigarettes) compared with studies of only smokers interested in cigarette cessation, study design, population, comparison group, control variables, time of exposure assessment, biochemical verification of abstinence, or definition of e-cigarette use. This result indicates that the overall conclusion that e-cigarettes are associated with less smoking cessation is not an artifact of the study design methods.

**Figure 3:**
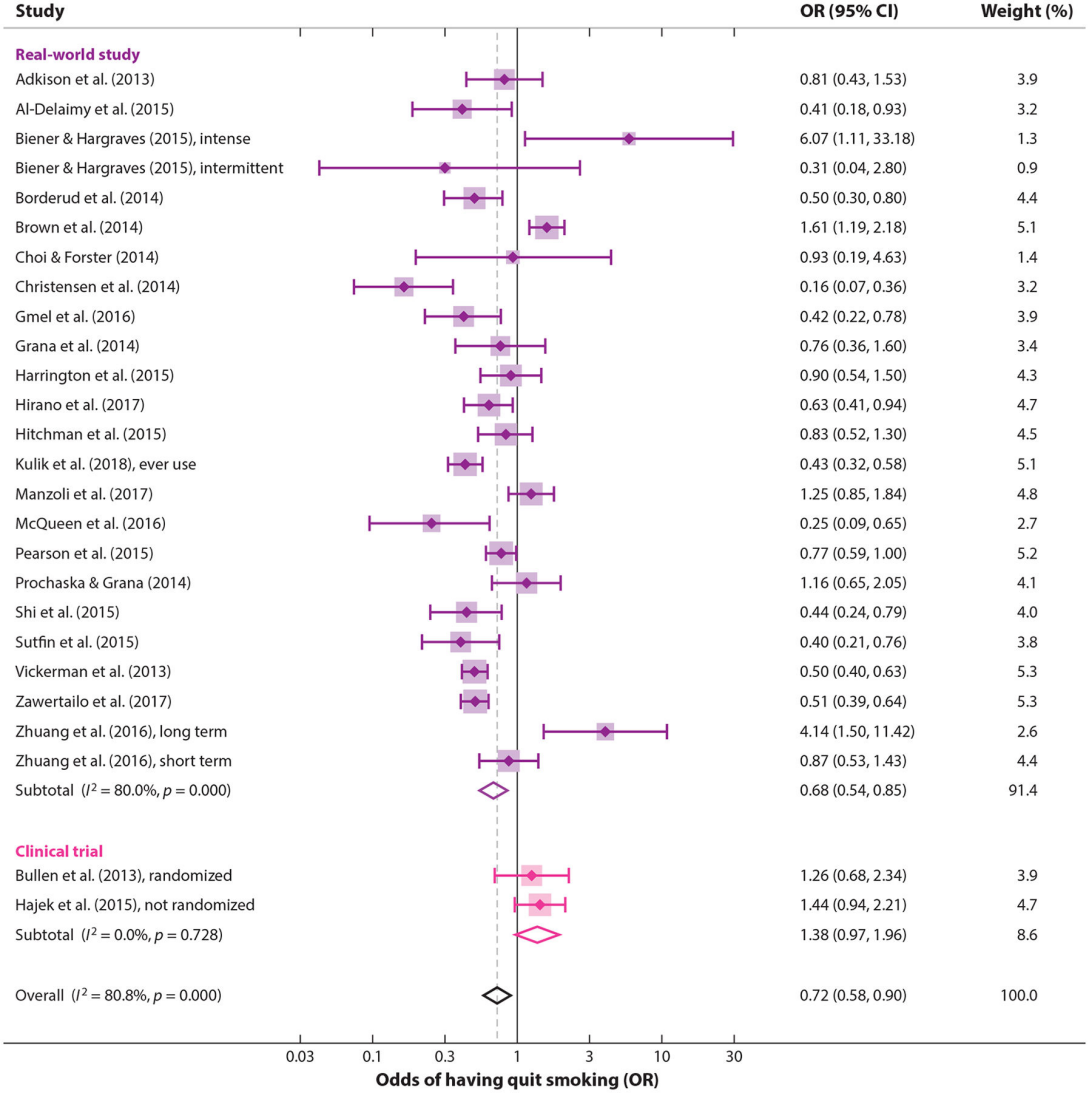
Smokers who use e-cigarettes are significantly less likely to have stopped smoking than smokers who do not use e-cigarettes, with the odds of quitting smoking depressed by 27%. Citations for studies: 2, 4, 19, 21, 22, 29, 30, 48, 54, 57, 62, 63, 75, 81, 86, 100, 104, 115, 124, 138, 147, 149, 151. Note: Weights are from random effects analysis. Abbreviations: CI, confidence interval; OR, odds ratio.

Between April 2015 and June 2017, seven more studies were published on the association between using e-cigarettes and quitting cigarettes (48, 62, 75, 81, 147, 149, 151). Updating the Kalkhoran & Glantz meta-analysis (70) to include these studies only slightly changed the pooled estimate of the effect (0.73, 95% CI 0.59–0.92) (Figure 3). The overall conclusion that smokers who used e-cigarettes were significantly less likely to stop smoking cigarettes remained.

Four studies (19, 63, 147, 151) did find significantly increased quitting among some e-cigarette users, suggesting that specific use patterns may be important. One study (19) found that intermittent e-cigarette users (more than once or twice but less than daily use) were less likely to quit smoking one year later than none-cigarette users, but those who had used e-cigarettes daily for at least one month were significantly more likely to quit cigarettes. Another study (63) found that all “cig-alike” users and nondaily tank system users had lower odds of quitting cigarettes, whereas daily tank system users were significantly more likely to quit. The third study (151) found that short-term e-cigarette use was not associated with a lower rate of smoking cessation, but long-term use was. The fourth study (147) found higher quitting smokers specifically using e-cigarettes as part of a quit attempt in countries with permissive e-cigarette policies (United States and United Kingdom) than those in countries with restrictive policies (Canada and Australia). In contrast, in the European Union (including Great Britain, specifically), a study of the relationship between e-cigarette use and having stopped smoking found less quitting among smokers who used e-cigarettes (75).

These results suggest that e-cigarettes are contributing to the tobacco epidemic by attracting smokers who are interested in quitting but reducing the likelihood of those smokers to quit successfully. This effect may be reflected in the fact that in 2015 the number of cigarettes consumed in the United States was higher than in 2014, the first time cigarette consumption increased since 1973 (139).

## HEALTH EFFECTS OF E-CIGARETTES

### Are “E-Cigarettes 95% Safer than Cigarettes”?

Influential health organizations in England, including Public Health England (85), the Royal College of Physicians (105), the Royal Society for Public Health (106), and the National Health Service (85, 96), have unequivocally stated that e-cigarettes are 95% safer than conventional cigarettes. This claim originated from a single consensus meeting of 12 people convened by D.J. Nutt in 2014 (97). They reached this conclusion without citing any specific evidence (32). The Nutt et al. paper did include this caveat: “A limitation of this study is the lack of hard evidence for the harms of most products on most of the criteria” (97, p. 224), which has generally been ignored by those quoting this report (85, 96, 105, 106).

A 2015 editorial in *The Lancet* (39) identified financial conflicts of interest associated with Nutt et al. (97), noting that “there was no formal criterion for the recruitment of the experts.” The Nutt et al. meeting was funded by EuroSwiss Health and Lega Italiana Anti Fumo (LIAF). EuroSwiss Health is one of several companies registered at the same address in a village outside Geneva with the same chief executive, who was reported to have received funding from British American Tobacco (BAT) for writing a book on nicotine as a means of harm reduction (66) and who also endorsed BAT’s public health credentials (127). Another of Nutt’s coauthors, Riccardo Polosa, was Chief Scientific Advisor to LIAF, received funding from LIAF, and reported serving as a consultant to Arbi Group Srl, an e-cigarette distributor. He also received funding from Philip Morris International (84, 129). Later in 2015, the *BMJ* published an investigative report (51) that raised broader issues surrounding potential conflicts of interest between individuals involved in the Nutt et al. paper. *BMJ* provided an infographic illuminating undisclosed connections between key people involved in the paper and the tobacco and e-cigarette industries as well as links between the paper and Public Health England via one of the coauthors. Even so, as of June 2017, the “95% safer” figure remains widely quoted, despite the fact that evidence of the dangers of e-cigarette use has rapidly accumulated since 2014. This new evidence indicates that the true risk of e-cigarette use is much higher than the “95% safer” claim would indicate.

### Cancer

Most discussion of the health effects of e-cigarettes has focused on cancer. As noted above, e-cigarettes deliver lower levels of carcinogens than do conventional cigarettes (50), and lower levels of carcinogens are found in the bodies of e-cigarette users than are found in smokers (114). While these observations suggest that e-cigarettes are likely less carcinogenic than conventional cigarettes, they do deliver carcinogens that can have effects at very low levels following repeat exposures (32). E-cigarettes deliver the tobacco-specific nitrosamine and potent lung carcinogen NNK [4-(N-methyl-N-nitrosoamino)-1-(3-pyridyl)-1-butanone, also known as nicotine-derived nitrosamine ketone] (50, 114). Some evidence indicates that the NNK dose-response curve for cancer is highly nonlinear, with substantial increases in risk at low doses (60). Known bladder carcinogens have been detected in the urine of e-cigarette users but not in nonusers (44). In addition, while nicotine is not a carcinogen, it does promote the growth of blood vessels that supply tumors and it speeds tumor growth (59).

**Figure 4:**
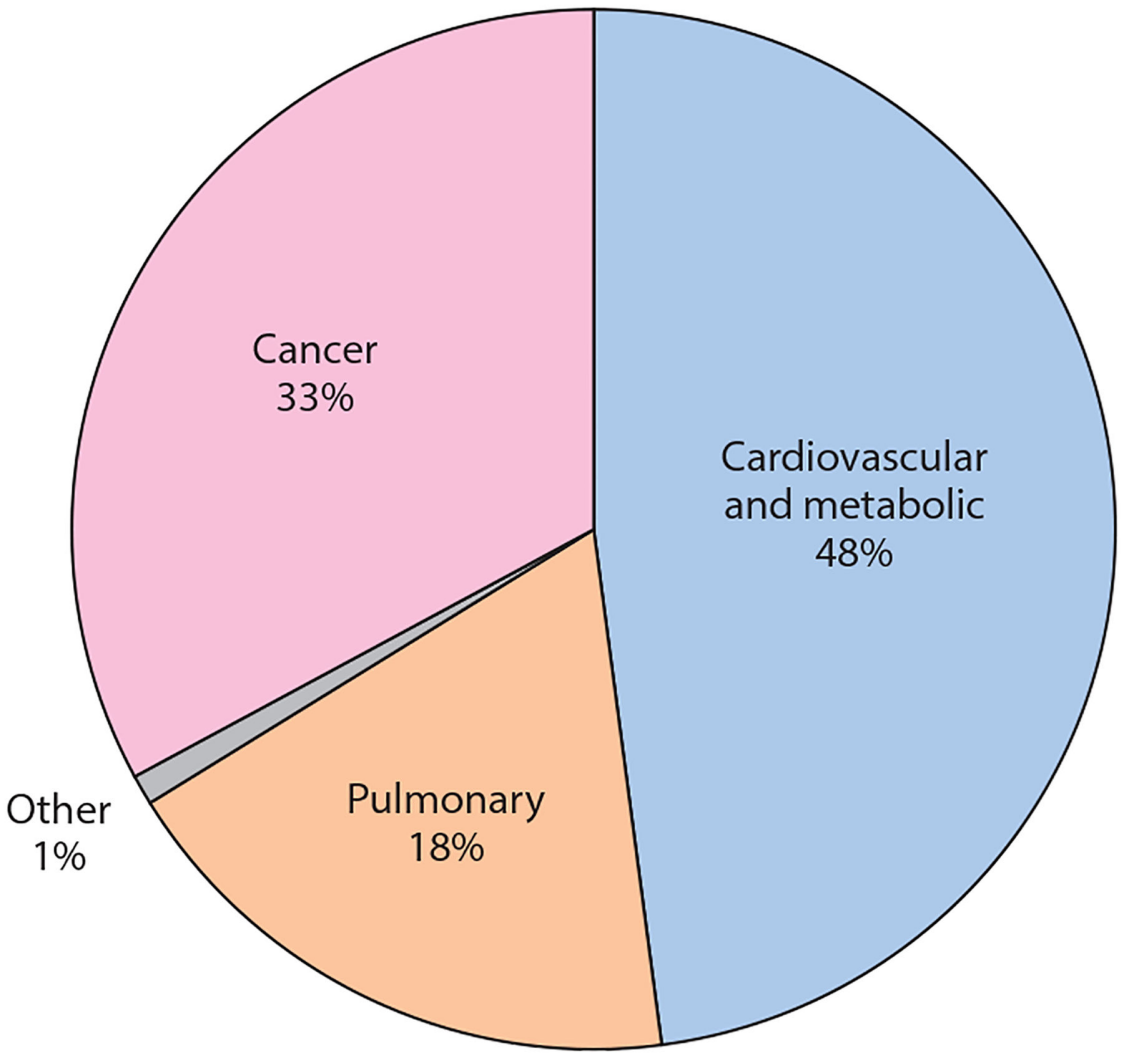
Between 1965 and 2015, active and passive smoking killed 21 million people. Although most discussion of smoking and disease focuses on cancer, cardiovascular disease and metabolic and noncancer pulmonary disease kill most smokers (134).

The fact is, however, cardiovascular and noncancer lung disease kill more smokers (135) than does cancer (Figure 4), which makes it important to assess the impact of e-cigarette use on these other diseases.

### Cardiovascular Disease

**Figure 5:**
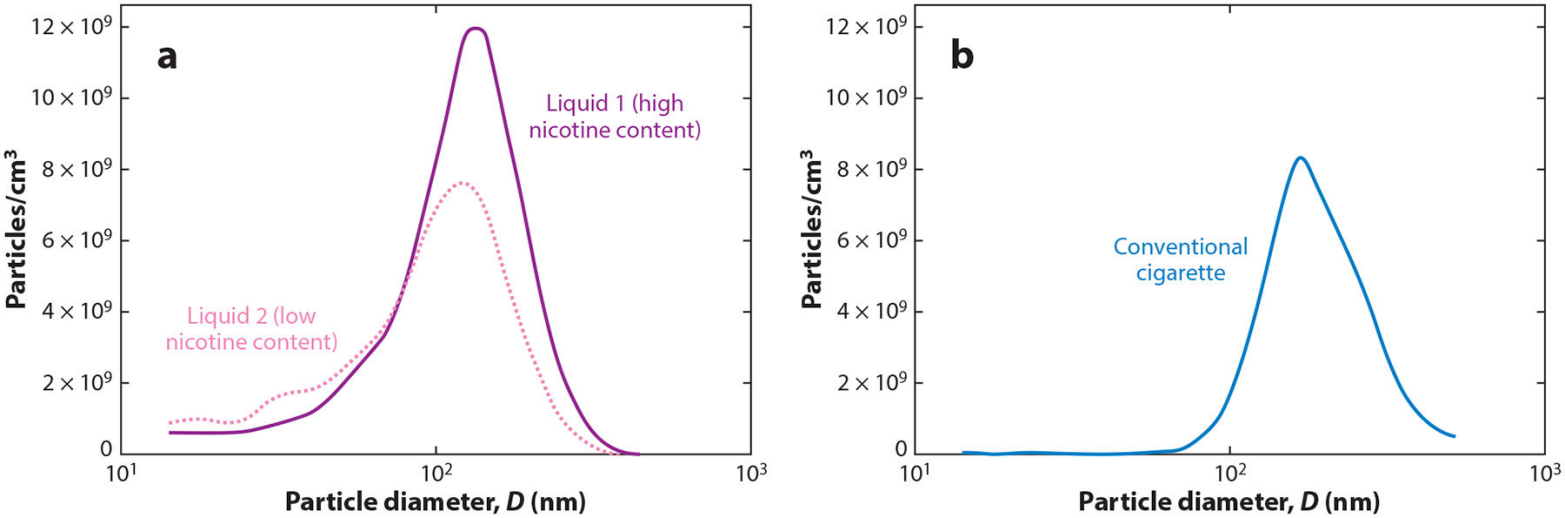
Particle number distribution from (*a*) mainstream aerosol in high and low nicotine content e-liquids and from (*b*) conventional cigarette as a function particle size (diameter, *D*). Adapted from Fuoco et al. (45) with permission from the publisher. Copyright © 2013 Elsevier Ltd.

E-cigarettes adversely impact the cardiovascular system (17, 113). Although the specific role of nicotine in cardiovascular disease remains debated (16, 17), nicotine is not the only biologically active component in e-cigarette aerosol. As noted above, e-cigarettes work by creating an aerosol of ultrafine particles to carry nicotine deep into the lungs. These particles are as small as—and sometimes smaller than—those in conventional cigarettes (45) (Figure 5). These ultrafine particles are themselves biologically active, trigger inflammatory processes, and are directly implicated in causing cardiovascular disease and acute cardiovascular events (101). The dose-response effect for exposure to particles is nonlinear, with substantial increases in cardiovascular risk with even low levels of exposure to ultrafine particles (101). For example, exposure to secondhand cigarette smoke has nearly as large an effect on many risk factors for cardiovascular disease and the risk of acute myocardial infarction as does being an active smoker (13). In addition, e-cigarettes expose users to acrolein and other aldehydes (17, 18). Like conventional cigarette smokers, e-cigarette users experience increased oxidative stress (26, 92) and increases in the release of inflammatory mediators (26, 61). E-cigarette aerosol also induces platelet activation, aggregation, and adhesion (64). All these changes are associated with an increased risk of cardiovascular disease.

These physiological changes are manifest in rapid deterioration of vascular function following use of e-cigarettes. E-cigarette and traditional cigarette smoking in healthy individuals with no known cardiovascular disease exhibit similar inhibition of the ability of arteries to dilate in response to the need for more blood flow (26). This change reflects damage to the lining of the arteries (the vascular endothelium), which increases both the risk of long-term heart disease and an acute event such as a myocardial infarction (heart attack) (141, 145, 146). Using e-cigarettes is also accompanied by a shift in balance of the autonomic (reflex) nervous system toward sympathetic predominance (26, 92), which is also associated with increased cardiac risk (56, 126).

The biological stresses that e-cigarette use imposes on the cardiovascular system are manifest as an increase in risk of acute myocardial infarction (125). A cross-sectional analysis of data in the US 2014 and 2016 National Health Interview Surveys revealed that daily e-cigarette use was associated with increased odds of having suffered a myocardial infarction (OR = 1.79, 95% CI 1.20–2.66; *p* = 0.004), controlling for conventional cigarette smoking, demographic characteristics (age, gender, body mass index, family income) and health characteristics (hypertension, diabetes, and hypercholesterolemia) (125). Significantly, the effect of using e-cigarettes on the odds of myocardial infarction approached what was found with conventional cigarette smoking (OR = 2.72, 2.29–3.24; *p <* 0.001) (125).

### Lung Disease

As with cardiovascular disease, evidence consistently indicates that exposure to e-cigarette aerosol has adverse effects on lungs and pulmonary function (31, 91). Repeated exposure to acrolein, which is produced by heating the propylene glycol and glycerin in e-liquids, causes chronic pulmonary inflammation, reduction of host defense, neutrophil inflammation, mucus hypersecretion, and protease-mediated lung tissue damage, which are linked to the development of chronic obstructive pulmonary disease (94). E-cigarette aerosol also exposes users to highly oxidizing free radicals (49). Animal studies have also shown that e-cigarettes increase pulmonary inflammation and oxidative stress while inhibiting the immune system (31).

Consistent with these experimental results, people who used e-cigarettes experienced decreased expression of immune-related genes in their nasal cavities, with more genes suppressed than among cigarette smokers, indicating immune suppression in the nasal mucosa (82). E-cigarette use upregulates expression of platelet-activating factor receptor (PAFR) in users’ nasal epithelial cells (90); PAFR is an important molecule involved in the ability of *S. pneumoniae*, the leading cause of bacterial pneumonia, to attach to cells it infects (adherence). In light of the immunosuppressive effects observed in nasal mucosa (82), there is concern that e-cigarette use will predispose users toward more severe respiratory infections, as has been demonstrated in mouse studies (67).

Given these effects, it is not surprising that e-cigarette use is associated with a doubling of the risk of symptoms of chronic bronchitis among US high school juniors and seniors (OR = 2.02; 95% CI 1.42–2.88) with higher risk associated with higher use; these risks persisted among former users (83). Similarly, current e-cigarette use was associated with an increased diagnosis of asthma among Korean high school students (adjusted OR = 2.74; 95% CI 1.30–5.78 among current e-cigarette users who were never cigarette smokers) (28). E-cigarette users were also more likely to have had days absent from school due to severe asthma symptoms.

### Summary of Health Effects

Although e-cigarettes deliver lower levels of carcinogens than do conventional cigarettes, and therefore may pose less cancer risk to users (albeit not zero cancer risk), they still expose users to high levels of ultrafine particles and other toxins that may substantially increase cardiovascular and noncancer lung disease risk. The similarities between the effects of e-cigarettes and those of conventional cigarettes on determinants of cardiovascular and lung disease make it likely that e-cigarettes will impose similar long-term cardiovascular and pulmonary risks as those associated with conventional cigarettes. Cardiovascular and noncancer pulmonary diseases account for about two-thirds of smokers’ premature deaths from tobacco-induced diseases (Figure 4), so it would not be surprising if e-cigarettes impose half (or more) of the overall long-term risks as those from conventional cigarettes.

## USE OF E-CIGARETTES IN SMOKEFREE ENVIRONMENTS

Using e-cigarettes in places where smoking is prohibited (e.g., workplaces, public places such as restaurants and bars, and otherwise smokefree homes) is one of the reasons that people use e-cigarettes (99, 116, 131). (This situation is changing as more places include e-cigarettes in their smokefree policies.) Even though e-cigarettes do not produce any sidestream smoke (the smoke that comes off the lit end of a smoldering cigarette), they do pollute the air in the form of exhaled mainstream aerosol from people using e-cigarettes. Nicotine, ultrafine particles, and products of heating propylene glycol and glycerin are increased in the air where e-cigarettes are being used, although, as expected, at lower levels than produced by smoking the same number of conventional cigarettes (12, 34, 46, 112).

As with conventional cigarettes, however, when several people are using e-cigarettes indoors at the same time, the air can become polluted. For example, levels of fine particulate matter (PM_2.5_) in a large hotel event room (4,023 m^3^) increased from 2–3 μg/m^3^ to as high as 819 μg/m^3^ interquartile range: 761–975 μg/m^3^) when 59–86 people were using e-cigarettes (120). This level is comparable to a very (conventional tobacco) smoky bar or casino and dramatically exceeds the US Environmental Protection Agency annual time-weighted standard for PM_2.5_ of 12 μg/m^3^ (137).

Evidence has also shown that bystanders absorb nicotine when people around them use e-cigarettes at levels comparable with exposure to conventional cigarette secondhand smoke (12). In a study of nonsmokers living with nicotine e-cigarette users, those living with conventional cigarette smokers, or those living in homes where no one used either product, cotinine (a metabolite of nicotine) levels in bystanders’ urine were significantly elevated in both the people exposed to secondhand e-cigarette aerosol and those exposed to secondhand tobacco smoke compared with people living in aerosol- and smoker-free homes. Interestingly, the levels of elevated urinary cotinine in the two exposed groups were not significantly different (although the passive smokers had higher point estimates), despite the fact that the increase in air pollution in the smokers’ homes was much higher than in the e-cigarette users’ homes (geometric mean air nicotine concentrations of 0.13 μg/m^3^ in e-cigarette users’ homes, 0.74 μg/m^3^ in smokers’ homes, and 0.02 μg/m^3^ in the control homes).

On the basis of emerging evidence, in 2014 the American Industrial Hygiene Association (3, p. 2) concluded that “e-cigarettes are not emission-free and that their pollutants could be of health concern for users and those who are exposed secondhand. …[T]heir use in the indoor environment should be restricted, consistent with current smoking bans, until and unless research documents that they will not significantly increase the risk of adverse health effects to room occupants.” Similarly, in 2016 the American Society of Heating, Refrigeration and Air-Conditioning Engineers (ASHRAE) updated its standard for “Ventilation for Acceptable Indoor Air Quality” to incorporate emissions from e-cigarettes into the definition of “environmental tobacco smoke,” which is incompatible with acceptable indoor air quality (10, 11). As of April 2017, 12 US states and 615 localities had prohibited the use of e-cigarettes in venues in which conventional cigarette smoking was prohibited (7).

## POLICY ISSUES

Initial hopes that e-cigarettes would be both a less toxic competitor to conventional cigarettes and a help to people who attempt to quit smoking cigarettes (76) have not translated into real-world positive effects. Instead, e-cigarettes have simply become another class of tobacco products that are maintaining and expanding the tobacco epidemic.

As the major tobacco companies have moved into, and increasingly dominated, the e-cigarette market, they are dominating the political and policy-making environments just as they have in conventional cigarette policy making (33, 78). As they have done to influence tobacco control policies for conventional cigarettes (132), the large companies often try to stay out of sight and work through third parties that can obscure their links to the tobacco industry (33). The one difference from the historical pattern of industry efforts to shape tobacco policy from behind the scenes is that there are also genuine independent sellers of e-cigarettes and associated users (so-called vape shops) who are not necessarily being directed by the cigarette companies. These smaller operators are, however, losing market share to the big tobacco companies (89), and the real political power is now being exercised by the cigarette companies. The cigarette companies try to take advantage of the existence of independent players while acting through the industry’s traditional allies and front groups (33, 42).

Countries have reacted in a variety of ways to the introduction of e-cigarettes in their markets, ranging from no regulations to a ban on e-cigarettes. The Conference of the Parties to the World Health Organization Framework Convention (which does not include the United States) has generally taken a cautious approach to e-cigarettes (140) and has agreed that regulatory measures need to be implemented to, at a minimum, ensure that e-cigarettes do not worsen the tobacco epidemic (140). Because of these realities, e-cigarettes should be integrated into tobacco control policies at all levels of government.

To minimize deleterious health effects, we recommend the following measures:

- Prohibit the use of e-cigarettes anywhere that use of conventional cigarettes is prohibited, including in smokefree homes;
- Tax e-cigarettes at levels comparable to cigarettes;
- Include e-cigarettes in public education campaigns, particularly communicating the facts that they are not “harmless water vapor,” do pollute the air, are a gateway to conventional cigarettes, and are increasingly sold by the same multinational companies that sell conventional cigarettes;
- Prohibit the sale of e-cigarettes to anyone who cannot legally buy cigarettes or in any venues where the sale of conventional cigarettes is prohibited;
- Establish a minimum purchase age of 21;
- Subject e-cigarettes to the same marketing restrictions that apply to conventional cigarettes (including no television, radio, or outdoor advertising);
- Prohibit cobranding of e-cigarettes with cigarettes or marketing in a way that promotes dual use;
- Prohibit flavored e-cigarettes, particularly menthol, candy, fruit, and alcohol flavors;
- Prohibit claims that e-cigarettes are effective smoking cessation aids until e-cigarette companies provide sufficient evidence that, as actually used in the real world, e-cigarettes are effective for smoking cessation;
- Prohibit any health claims about e-cigarette products until and unless they are authorized by the appropriate regulatory agencies (the FDA in the United States) using scientific and regulatory standards that account for dual use and effects of e-cigarette use on depressing smoking cessation; and
- Establish quality standards for ingredients and functioning of e-cigarette devices.

Implementing these policies would reduce the likelihood that e-cigarettes will continue to expand and extend the tobacco epidemic.

## THE FUTURE

Because e-cigarettes have been on the market for only a few years, the long-term population health effects are not known. Nevertheless, it is already clear that e-cigarettes are prolonging and extending the tobacco epidemic by reducing smoking cessation and expanding the tobacco market by attracting youth who would otherwise be unlikely to initiate tobacco use with conventional cigarettes. On the basis of the short-term effects that have been identified to date, e-cigarettes likely have cardiovascular and noncancer lung disease risks similar to those associated with smoking conventional cigarettes. Under most reasonable alternative use pattern scenarios, this is a high enough risk to lead to a net population harm even if some smokers switch to e-cigarettes (47, 69, 80). To minimize harm, e-cigarettes as well as the timing and location of their promotion and use should be regulated like other tobacco products.

## Figures and Tables

**Table 1:** Types of e-cigarettes. Reproduced under the terms of the CC-BY-NC-ND license, Reference 53

Product	Description	Some brands
Disposable e-cigarette 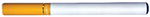	Cigarette-shaped device consisting of a battery and a cartridge containing an atomizer to heat a solution (with or without nicotine). Not rechargeable or refillable and is intended to be discarded after product stops producing vapor. Sometimes called an e-hookah.	NJOY OneJoy, Aer Disposable, Flavorvapes
Rechargeable e-cigarette 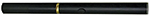	Cigarette-shaped device consisting of a battery that connects to an atomizer used to heat a solution typically containing nicotine. Often contains an element that regulates puff duration and/or how many puffs may be taken consecutively.	Blu, GreenSmoke, EonSmoke
Pen-style, medium-sized rechargeable e-cigarette 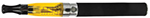	Larger than a cigarette, often with a higher-capacity battery, may contain a prefilled cartridge or a refillable cartridge. Often come with a manual switch allowing the user to regulate length and frequency of puffs.	Vapor King Storm, Totally Wicked Tornado
Tank-style, large-sized rechargeable e-cigarette 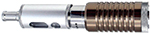	Much larger than a cigarette with a higher-capacity battery and typically contains a large, refillable cartridge. Often contains manual switches and a battery casing for customizing battery capacity. Can be easily modified.	Volcano Lavatube
